# Insight the C-Site Pocket Conformational Changes Responsible for Sirtuin 2 Activity Using Molecular Dynamics Simulations

**DOI:** 10.1371/journal.pone.0059278

**Published:** 2013-03-20

**Authors:** Sugunadevi Sakkiah, Mahreen Arooj, Guang Ping Cao, Keun Woo Lee

**Affiliations:** 1 Division of Applied Life Science (BK21 Program), Systems and Synthetic Agrobiotech Center (SSAC), Plant Molecular Biology and Biotechnology Research Center (PMBBRC), Research Institute of Natural Science (RINS), Gyeongsang National University, Jinju, South Korea; 2 School of Life Sciences, Gwangju Institute of Science and Technology, Gwangju, South Korea; 3 Steitz Center for Structural Biology, Gwangju Institute of Science and Technology, Gwangju, South Korea; University of Akron, United States of America

## Abstract

Sirtuin belongs to a family of typical histone deacetylase which regulates the fundamental cellular biological processes including gene expression, genome stability, mitosis, nutrient metabolism, aging, mitochondrial function, and cell motility. *Michael et. al.* reported that B-site mutation (Q167A and H187A) decreased the SIRT2 activity but still the structural changes were not reported. Hence, we performed 5 ns molecular dynamics (MD) simulation on SIRT2 Apo-form and complexes with substrate/NAD^+^ and inhibitor of wild type (WT), Q167A, and H187A. The results revealed that the assembly and disassembly of C-site induced by presence of substrate/NAD^+^ and inhibitor, respectively. This assembly and disassembly was mainly due to the interaction between the substrate/NAD^+^ and inhibitor and F96 and the distance between F96 and H187 which are present at the neck of the C-site. MD simulations suggest that the conformational change of L3 plays a major role in assembly and disassembly of C-site. Our current results strongly suggest that the distinct conformational change of L3 as well as the assembly and disassembly of C-site plays an important role in SIRT2 deacetylation function. Our study unveiled the structural changes of SIRT2 in presence of NAD^+^ and inhibitor which should be helpful to improve the inhibitory potency of SIRT2.

## Introduction

The posttranslational modification regulates a number of cellular processes including the modulation of DNA accessibility, replication and repair, acetylation and deacetylation of proteins. Acetylated histones are generally correlated well with gene activity, whereas deacetylated histones are associated with chromatin structure that is less accessible to transcriptional activation [1]. One of the best post translation modifications is the cleavage of acetyl group from α-amino group of lysine residues in histone tails of nucleosomes catalyzed by histone deacetylases (HDACs) family. So far, four different classes of HDACs have been identified in humans and ramifications into two groups based on its mechanism (i) the zinc-dependent or classical HDACs: HDAC 1, 2, 3 and 8 are homologues of reduced potassium dependency (Class I), Class II includes six subtypes and divided into two subclasses, class IIa (HDAC 4, 5, 7, 9) and Class IIb (HDAC 6,10) and HDAC 11 comes under Class IV (ii) the nicotinamide adenine dinucleotide (NAD^+^) dependent Sir2 proteins (sirtuins, Class III HDAC) [2]. The HDACs and histone acetyltransferases (HATs) regulate the activity of non-histone protein targets such as p53 (deacetylated by HDAC1) [3], [4] or α-tubulin (by HDAC6 and Sirt2) [5].

**Figure 1:**
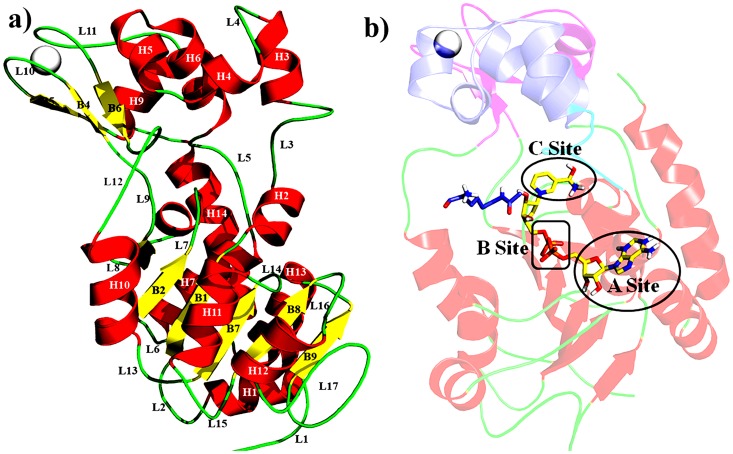
SIRT2 Structure properties a) Secondary structural detail, and b) Helical Domain (blue), Zinc binding domain (magenta), Zinc (gray) and Large domain (red).

Sirtuins are a class of NAD^+^-dependent deacetylase protein and ADP-ribose-transferase enzymes homologous to yeast Sir2 protein [6], [7] and evolutionally conserved from bacteria to mammals [8]. Sirtuins have been implicated in the control of diverse biological processes, such as apoptosis, aging, transcriptional silencing, chromosomal stability, neurodegeneration, cell cycle progression, autophagy, growth suppression, inflammation, stress response and overall cellular metabolism [9]–[13]. In human, seven distinct NAD^+^-dependent Sirtuin enzymes (SIRT1-7) were reported which are involved in the regulation of neuronal survival. The seven enzymes (SIRT1-7) shared conserved catalytic core domain of ∼275 amino acids but differ in their N- and C- terminal. Indeed, SIRT1, present in nucleus, has been reported to protect against neuronal death. Mostly SIRT2 present in the cytoplasm and nucleus where it associates with microtubules and deacetylates α-tubulin [14], [15] and promotes neurodegeneration. SIRT3, SIRT4 and SIRT5, localized in mitochondria, play a role in energy metabolism and responses to oxidative stress [16]. A growing body of evidence implicates that SIRT1 and SIRT2 are important regulators of neurodegeneration [13], [17]. Recent studies find that SIRT2, a second member of the NAD^+^-dependent HDAC family, which is most widely known for its ability to interact with α-tubulin [14]. The structure of SIRT2 catalytic domain consists of large and small domains (Figure. 1). The large domain contains a typical Rossmann fold, comprises of 6 parallel ß-sheets surrounded by 6 α-helices. The small domain was classified into Zinc (Zn) binding and helical domains. The Zn binding domain consists of 3 anti-parallel ß-sheets, one α-helix and Zn^2+^ was anchored by 4 conserved Cysteine residues (C195, C200, C221, and C224) in all classes of Sir2-like enzymes and the helical domain comprised of 4 helices, 2 short and 2 long helices. The cofactor-binding (NAD^+^) pocket was divided into three regions: adenine ribose moiety of NAD^+^ is bound in A-site, nicotinamide ribose moiety in B-site and C-site, nicotinamide is located deep inside the catalytic pocket [18]. Small molecules that can modulate sirtuin activity have been shown to have potential in treating cancer [19], [20], Parkinson’s disease [21], obesity and diabetes [22]–[25], aging and aging-related diseases [26] as well as it is a putative therapeutic target for affecting Huntington disease (HD) mediated transcriptional dysregualtions.

*Michael et. al.* experientially proved that the mutations of Q167A and H187A reduced the SIRT2 deacetylation activity and strongly suggest that these two residues are key player in the catalysis [18], but fail to produce the structural changes responsible for activity deterioration. The molecular dynamics (MD) simulation is a valuable tool to investigate a wide range of conformational change and motional phenomena in biomolecular systems such as proteins and nucleic acids [27]. MD simulation is an alternative and very efficacious technique through which one can gather information regarding the conformational changes of the protein due to the substrate or inhibitor binding. MD simulation is widely used to obtain information based on the time-dependent conformational change that is experimentally inaccessible. In this work, the MD simulation method was utilized to examine the structure and functional effect of wild type (WT) and SIRT2 mutant (Q167A and H187A) in B-site. Totally, nine independent MD simulations were carried out to obtain the equilibrium trajectories and detailed analysis of these trajectories suggested that the conformational rearrangement will be responsible to trigger and suppress the SIRT2 activity.

## Methods and Materials

### Molecular Docking Study

Due to the absence of experimentally determined SIRT2 complexes, molecular docking technique was employed to obtain the complex structures. The atomic coordinates of Apo SIRT2 under the accession code 1J8F [18] was taken from Protein Data Bank (PDB) [28]. The 2D form of acetylated lysine (substrate), co-factor (NAD^+^), and inhibitor were sketched using ChemSketch v12 and converted into 3D format with the help of Discovery Studio (DS) v2.5. *LigandFit* was used to understand the hydrogen bond, hydrophobic interactions between the SIRT2 and small molecules (substrate, NAD^+^, and inhibitor). Only the water present in the active was taken for the docking process and the remaining water molecules were removed. The hydrogen atoms were added by applying CHARMm [29] force field to neutralize the receptor, to attain its suitable orientation in energy minimization. *LigandFit* have two methods to find the binding sites (i) protein-ligand complex, the sole binding site can be found using the ‘docked ligand’ mode and (ii) absence of protein-ligand complex (known shape only), employs a cavity finding algorithm for detecting invaginations of possible candidate active site regions in protein.

To obtain the most stable energy-minimized conformation of small molecules, maximum number of 255 conformations were generated for each by applying Poling algorithm [30] using the *Best Conformation* with a constraint of energy value greater than 20 kcalmol^−1^ from the global minimum. We generated the SIRT2 complexes by docking NAD^+^, acetylated lysine, and inhibitor into its suitable binding site. The resultant docked complexes with substrate/NAD^+^, inhibitor, and Apo-form were used as a starting structure for MD simulations. The two mutants were constructed using the interactive DS by replacing Q167 and H187 in the WT with alanine.

### Molecular Dynamics Simulation

MD simulation was performed to gain insight into processes on an atomistic scale and also to analyze the dynamics behavior of protein and small molecule in aqueous solution. The classical MD simulations were performed using GROMACS 3.3 software package [31]–[33] by applying leap-frog integration steps to solve the equations of motion. Herein we used 9 systems for MD simulations study, each of 5 ns for WT, Q167A and H187A, Apo-form as well as with substrate/NAD^+^ and inhibitor. Since the topology of substrate/NAD^+^ and inhibitor was not available for GROMOS96 [34], [35] force field, we used the PRODRG server [36] to generate a set of parameters for bonds and angles based on the similar force field. No new atom types are included for substrate/NAD^+^ and inhibitor so that the atom charges and force constants are defined in the GROMOS96 force field. The SP3 [37] water model was used to create aqueous environment and the periodic boundary conditions were applied in all directions. The system was solvated with SPC water molecules. Before the MD simulation, an energy minimization was performed for all the nine systems (WT and mutants Apo-form, presence of substrate/NAD^+^ and inhibitor) to remove the poor van der Waal contacts in initial geometry. After the minimization process, two stages of equilibration were conducted: (i) 500 ps MD simulation of protein non-hydrogen atoms with positions restrained to allow the formation of solvation layers and (ii) another of 2 ns without positional restriction [38], [39]. The second step is important for the systems to accommodate the thermodynamics conditions imposed in simulations. After the equilibration stages, nine systems were subjected to 5 ns MD simulations for further analysis. All MD simulations applied the constraints algorithms SETTLE [40] and LINCS [41] for a time step of 2 fs. The LINCS algorithm was used to constrain all covalent interaction in non-water molecules, while the SETTLE algorithm was used to constrain bond lengths and angles in water molecules. A twin range cutoff was set for long-range interactions: 0.9 nm for van der Waals and 1.4 nm for electrostatic interactions. For coulomb interactions, the reaction field correction term [42] was employed, with a dielectric constant set to 0.8 [43]. Cutoff values of 1.4 and 0.8 Å were used for van der Waals and coulomb interactions, respectively. A periodic boundary condition in the isobaric-isothermal (NPT) ensemble with a constant temperature of 300 K and pressure at 1 atm by using Berendsen weak coupling [44] time of 0.2 ps was employed to control the temperature. Finally, the production run was performed for 5 ns and non-bonded interactions were calculated as well as the particle mesh Ewald method [45] was applied to treat the long-range electrostatic interactions. We performed MD simulations at 300 K on the Apo-form and complexes to investigate the dynamic nature of the interactions between the protein and substrate/NAD^+^ as well as with inhibitor. The average structure was calculated from the last 2 ns of the WT as well as mutants for subsequent structural analyses. All molecular structures were inspected using the VMD [46] and PyMol [47].

## Results and Discussions

### Molecular Docking

The molecular docking approach is one of the most reputable methods in drug discovery process to find the critical interactions between protein-ligand and protein-protein complexes. Docking accuracy was measured by the relative true binding mode of small molecules in receptor active site which determine the quality of docking methodology. Until now there is a lack of SIRT2 complex structures, hence molecular docking was carried out using the SIRT2 Apo-form as a receptor to identify the suitable active site as well as the binding orientation of substrate/NAD^+^ and inhibitor. The apo-form of SIRT2 (PDB: 1J8f) was selected as receptor and the NAD^+^ and acetylated lysine were docked in the suitbale binding pocket which exit between the small and large domain. The inhibitor was docked in the “C” site of NAD binding pocket which are mainly responsible for the deacetylase process of SIRT2 [48], [49]. The docking process saved top 25 conformations for each molecule (NAD+, acetylated lysine and inhibitor) based on the dock score value after the energy minimization using the *smart minimizer* method which begins with steepest descent algorithm and followed by conjugate gradient method. Among the 25 conformation, the complexes which show a high docking score was selected as initial SIRT2-complex structures for MD simulations. Based on the literatures, NAD binding pocket residues of SIRT2 was confirmed by multiple sequence analysis of sirtuin family. The NAD+ and the inhibitor binding site were well defined and reported in many literatures. Hence to further validate the complex structures, hydrogen bond interactions between the SIRT2 and the NAD+ and inhibitor were analyzed (Table 1). Finally, the observed binding site of SIRT2 was verified by comparing the volume occupied by NAD^+^ in the complex structure of yeast sirtuin (PDB ID: 1SZC) [50]. For inhibitor, Sir2Af2 bound with NAD, ADPribose and nicotinamide was used as a reference structure (PDB ID: 1YC2) [51] to identify the perfect binding site. The comparison of NAD+ and nicotinamide binding site in the crystal structure of yeast and Af2 Sirtuin shows a similar binding mode, respectively. The docked substrate/NAD^+^ and inhibitor are well placed in the SIRT2 active site and showed all necessary interactions which are reported in the literatures [50], [51]. Finally, the best SIRT2 complexes (File S1) were subjected to MD simulation to observe its structural changes due to the binding of substrate/NAD^+^ and inhibitor.

**Table 1:** Active site residues in Sirutin 2.

NAD Binding Site	Adenosine Binding Site					Ribose Binding Site		Nicotinamide Binding Site				
Literature Report	N286	E288	R97	A85	Q167	H187	S88	H149	F96	N168	I169	D170
Docking Result												
WT-NAD	N286	−	−	A85	Q167	H187	−	−	−	N168	I169	D170
Q167A-NAD	N286	−	−	A85	−	−	−	−	−	N168		D170
H187A-NAD	N286	−	−	A85	Q167	−	−	−	−	N168	I169	D170
WT-Inhibitor	−	−	−	−	Q167	−	−	−	−	−	I169	−
Q167A-Inhbitor	−	−	−	−	A167	H187	−	−	−	−	I169	−
H187A-Inhibitor	−	−	−	−	Q167	−	−	−	−	−	I169	−

### Molecular Dynamics Simulation

The objective of our MD simulation study was to explore the assembly of C-site will enhance the deacetylation process in SIRT2. In order to understand the internal motions and conformational changes of Apo-form and SIRT2 complexes, WT and mutants, namely, H187A and Q167A, the 5 ns time scale MD simulations were performed and analyzed. The summary of data obtained for 9 SIRT2 systems are presented in Table 2. The systems stability and fluctuation of the residues were confirmed by calculating root mean square deviation (RMSD) and root mean square fluctuation (RMSF) of Cα atom, respectively.

**Table 2:** Summary of nine model systems details for molecular dynamics simulations calculations.

System*	Protein Atoms	Water Molecules	Na+ Ions
WT_Apo	3038	20486	4
WT_Sub	3110	20443	2
WT_Inhi	3071	20461	4
Q167A_Apo	3032	20495	4
Q167A_Sub	3106	20465	1
Q167A_Inhi	3065	20468	4
H187A_Apo	3032	20496	4
H187A_Sub	3106	20444	1
H187A_Inhi	3065	20467	4

* WT_Apo: Wild type Apo-form, WT_Sub: wild type in presence of NAD and acetylated lysine; WT_Inhi: wild type in presence of inhibitor; Q167A: Glutamine mutate to Alanine Apo-form, Q167A_Sub: Glutamine mutate to Alanine in presence of NAD and acetylated lysine; Q167A_Inhi: Glutamine mutate to Alanine in presence of inhibitor; H187A: Histidine mutate to Alanine Apo-form, H187A_Sub: Histidine mutate to Alanine in presence of NAD and acetylated lysine; H187A_Inhi: Histidine mutate to Alanine in presence of inhibitor.

### Stability and Flexibility of SIRT2 Structures during Molecular Dynamics Simulation

**Figure 2:**
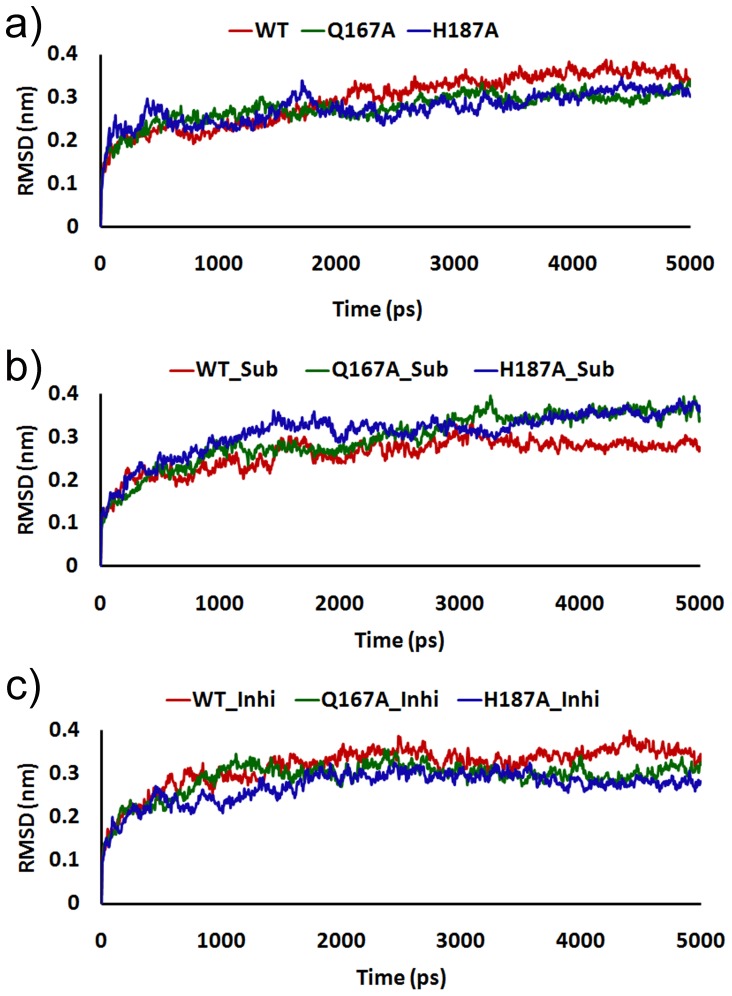
Root mean square deviation (RMSD) of Cα atoms. a) Apo_form, b) SIRT2 bound with substrate/NAD^+^, and c) SIRT2 with inhibitor.

An initial evaluation of structural drift provided the stability of protein in 9 systems by the RMSD analyzes of Cα atoms with respect to the function of time for WT, Q167A and H187A. The RMSD plots of WT and mutants during 5 ns simulation demonstrated the stability of proteins. The RMSD value of WT Apo-form was varies between 0.32 and 0.33 nm, but in case of both mutants it showed the variations of 0.28–0.30 nm (Figure. 2). These data revealed that the stability of Apo-form increased upon mutations. The inhibitor complexes of WT also have shown greater deviation when compared with both mutants but in case of substrate/NAD^+^ the mutant complexes shows much deviation than WT (Figure. 2). From the 5 ns production run, last 2 ns simulation of whole system is fairly stable indicated by small magnitude of RMSD of the Cα atom which confirmed the protein stability. Hence, the representative structures for 9 systems were obtained from last 2 ns of MD simulation trajectory used for further analyses.

**Figure 3:**
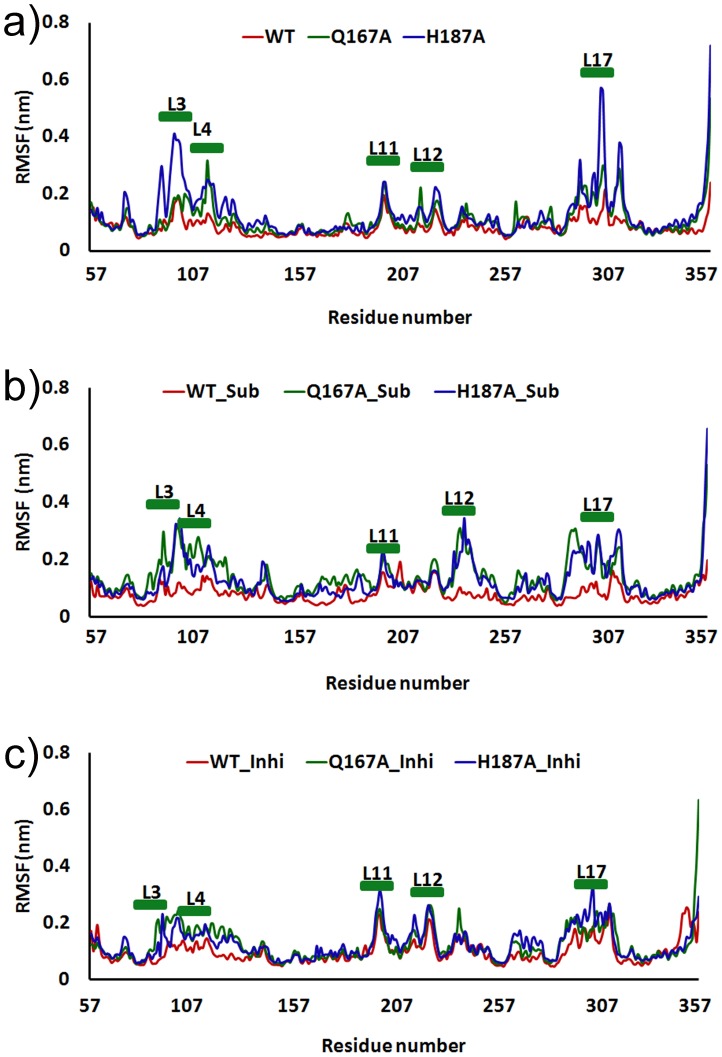
Root mean square fluctuation (RMSF) of Cα atoms. a) Apo_form, b) SIRT2 bound with substrate/NAD^+^, and c) SIRT2 with inhibitor.

The relative flexibility of each system was characterized by plotting RMSF for both WT and mutants (Figure. 3). The WT_Apo shows the values less than 0.2 nm indicates the minimum fluctuation. Q167A_Apo shows the similar fluctuation compared with WT_Apo except L4, L11, and L17 where there is slight increase (>0.2 nm). In case of H187A, the plot shows significantly larger fluctuation (∼0.4 nm) in the conserved loops (Figure. 3a). In presence of substrate/NAD^+^ and inhibitor binding all the systems were stabilized and shown the similar fluctuation.

### Conformational Changes in SIRT2 Wild Type Compared with Mutants

#### In presence of substrate/NAD^+^

**Figure 4:**
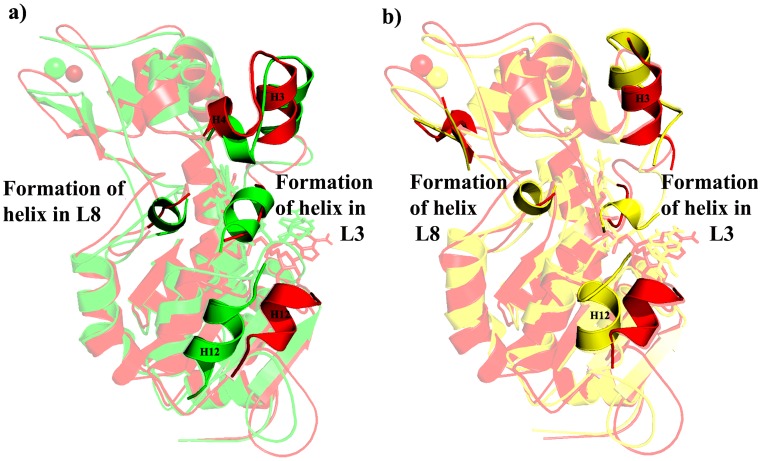
Structural comparison of SIRT2 in presence of substrate/NAD^+^ a) WT and Q167A and b) WT and H187A. Red, green, and yellow indicates WT, Q167A, and H187A, respectively. NAD was shown in stick.

Comparing the domains present in WT and Q167A, small shift in the helical and Zn binding domains and H12 present in the large domain shows a considerable deviation albeit there is not any considerable secondary structure change in large and small domains. Interestingly part of the two conserved loops (L3 and L8) which connects the small and large domains was changed into helix (Figure. 4a).

In case of H187A, there is a small helix formation in L3, L8 as well as there is a considerable deviation in the H3 present in the helical domain. The three anti-parallel β-sheets were changed into loop which indicated a distortion of Zn binding domain (Figure. 4b). Considerable changes were observed in the H12 present in the large domain. Here also there is a formation of helix in the two conserved loops (L3 and L8) connects the small and large domains.

#### In presence of inhibitor

**Figure 5:**
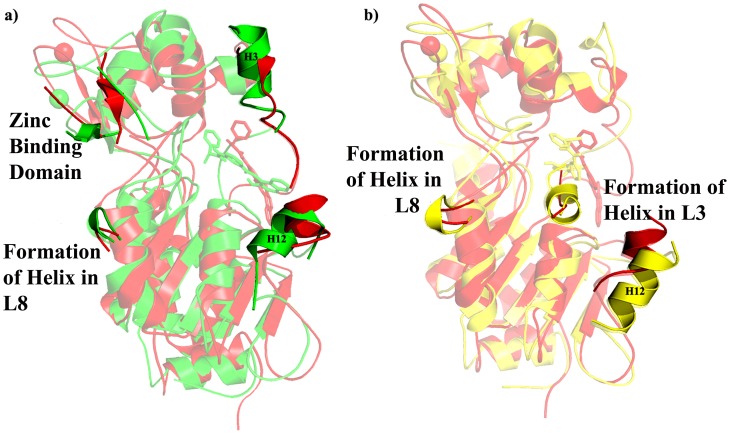
Structural comparison of SIRT2 in presence of inhibitor a) WT and Q167A and b) WT and H187A. Red, green, and yellow indicates WT, Q167A, and H187A, respectively. Inhibitor was shown in stick.

In Q167A, Zn binding domain was distorted due to the change of secondary structure conformation from anti-parallel ß-sheet into loop. The H3 which was partial distortion in WT has not shown any changes due Q167A. There was a helix formation in the conserved L8 and H12 was partially distorted and shifted above in Q167A. In H187A, H3 was able to distort as in WT but there is not any changes in Zn binding domain. Formation of helix in the conserved loops L3 and L8 and there was an extension of H12 was observed in large domain (Figure. 5).

### Comparing the Structural Displacement in NAD^+^ Binding Pocket in Wild Type and Mutants

#### In presence of substrate/NAD^+^

The WT, NAD^+^ interacts with most of the critical residues present in A, B and C-site of SIRT2 and forms a strong π-π stacking interaction between the nicotinamide of NAD^+^ and phenyl group of F96. The adenosine moiety of NAD^+^ shows good interactions with A85, G86, R97, N286, K287, E288, E323 and C324 which are present in A-site.

**Figure 6:**
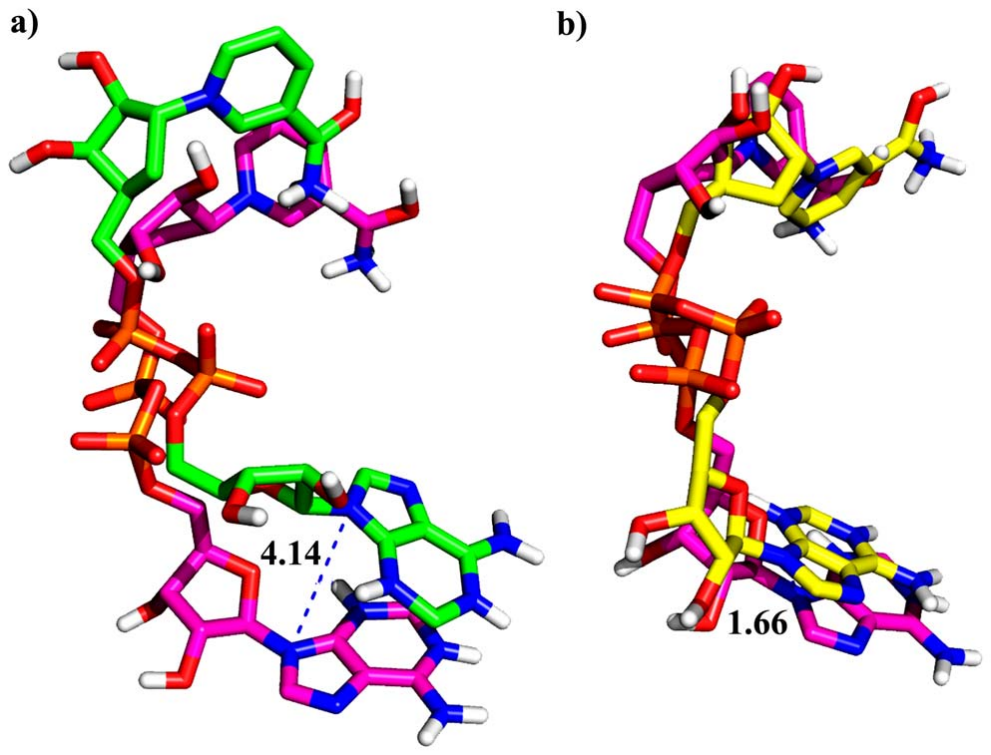
Superimpose of binding orientation of NAD^+^ in SIRT2 active site. a) WT with Q167A, deviation of N9A of adenine was 4.14 Å and b) WT and H187A deviation of N9A of adenine was 1.66 Å. WT-magenta, Q167A-green, and H187A-yellow.

Comparing the binding mode of NAD^+^ in WT and mutants showed the shift between N9A in NAD^+^ for Q167A (0.41 nm) and H187A (0.16 nm) in the adenosine moiety (Figure. 6). Hence we compute the Cα distance to find the deviation of each residues present in A-site. In Q167A, the A85 (Cα: 0.233 nm), R86 (Cα: 0.33 nm, CZ: 0.96 nm), N286 (ND2∶0.31 nm), K287 (Cα: 0.28 nm), and E288 (Cα: 0.51 nm, CD: 0.63 nm) shows a deviation compared with WT. In case of H187A, the R86 shows much deviation when compared to other A-site residues such as A85 (Cα: 0.28 nm), N286 (Cα: 0.25 nm, N: 0.25 nm), K287 (Cα: 0.24 nm) and E288 (Cα: 0.25 nm and CD: 0.25 nm). Among all the residues in A-site, R97 and E288 show a considerable deviation in both mutations.

The residues 167, 187 present in B-site which helps the nicotinamide part of NAD^+^ to enter into the C-site by making interactions with phosphate groups of NAD^+^ in WT. The Cα distance of residues 167 and 187 was calculated between the WT and mutants; the mutation of Q167 into A shows a Cα deviation of 0.16 nm (Q167A). Due to the mutation of Q167A, H187 showed a remarkable deviations of 2.56 Å in Cα and 4.72 Å in side chain. In H187A, the Cα distance between H187 (WT) and A187 in H187A shows a deviation of 2.06 Å but the Q167 residue not shows a deviation as expected.

**Figure 7:**
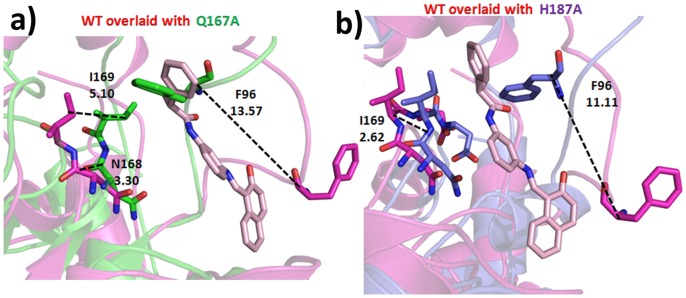
Comparison of C-site deviation due to the presence of NAD^+^. a) WT_Sub and Q167A, and b) WT_Sub and H187A. The distance are represented in Å.

The C-site residues such as S88, F96, H149, N168, D169, and I170 interacts with nicotinamide part of NAD^+^ which helps in the polarization and hydrolysis NAD glycosidic bond. In Q167A, the Cα deviation of 2.81 Å for S88, 4.34 Å for F96, 4.0 Å for N168, 3.41 Å for I169, and D170 (2.36 nm) (Figure. 7a). In case of H187A, F96 (Cα 0.26 nm, CZ 6.61 Å), N168 (Cα 2.82 Å, I169 Cα 3.38 Å), D170 (Cα 2.1 Å) deviation was observed when compared with WT (Figure. 7b). In both type of mutations we observed that the F96 has shown a huge deviations when compared with WT.

**Figure 8:**
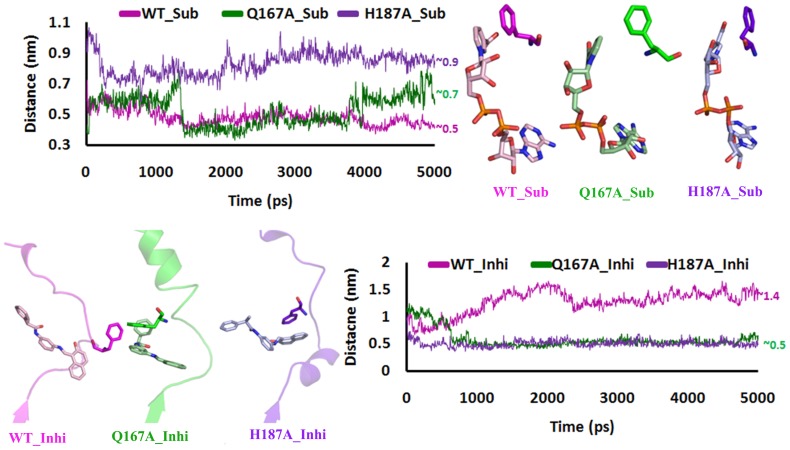
Distance between phenyl ring of F96 and phenyl group of NAD and inhibitor was plotted as a function of time in presence of NAD and inhibitor.

In WT, after 3 ns the phenyl ring of F96 moves closely to NAD^+^ from 0.61 to 0.43 nm which is in the range of the aromatic (π-π) interaction. In Q167A and H187A the distance was gradually increased to 0.64 and 0.8 nm, respectively (Figure. 8). Hence due to these mutations the π-π stacking interaction was lost. This indicates that F96 forms a tight stacking with NAD^+^ which is one of the critical interactions in SIRT2 mechanism and plays a vital role in the formation of **C**-site pocket. The π-π stacking interaction may be one of the reasons to reduce the flexibility of substrate/NAD^+^ complex and made the structure more stable throughout the MD simulations.

#### In presence of inhibitor

In Q167A, A-site residues have shown only a side chain deviation of 9 Å, 3.47 Å, 3.6 Å, 3 Å for R41, E267, K231, and E232, respectively. In H187A, only R41, K231, and E232 shows a side chain deviations of 8.07 Å, 6.48 Å, and 3.98 Å, respectively. In case of B-site, both the mutations does not shown substantial changes in residue 167 but shows a Cα deviation of 2.56 Å and 2.06 Å in Q167A and H187A. In both mutations, the C-site F96 was not able to move away from its original position as we observed in WT. One of the reasons for F96 rigidity in Q167A was the disability of distortion of H3 present in the helical domain. In case of H187A, albeit there was a distortion in H3 but this was moved upwards when compared with WT. The Cα distance of F96 between WT and Q167 was 13. 57 Å and WT and H187A shows a distance of 11.11 Å.

### Hydrogen Bonds Analysis between NAD^+^ and Inhibitor in SIRT2

**Figure 9:**
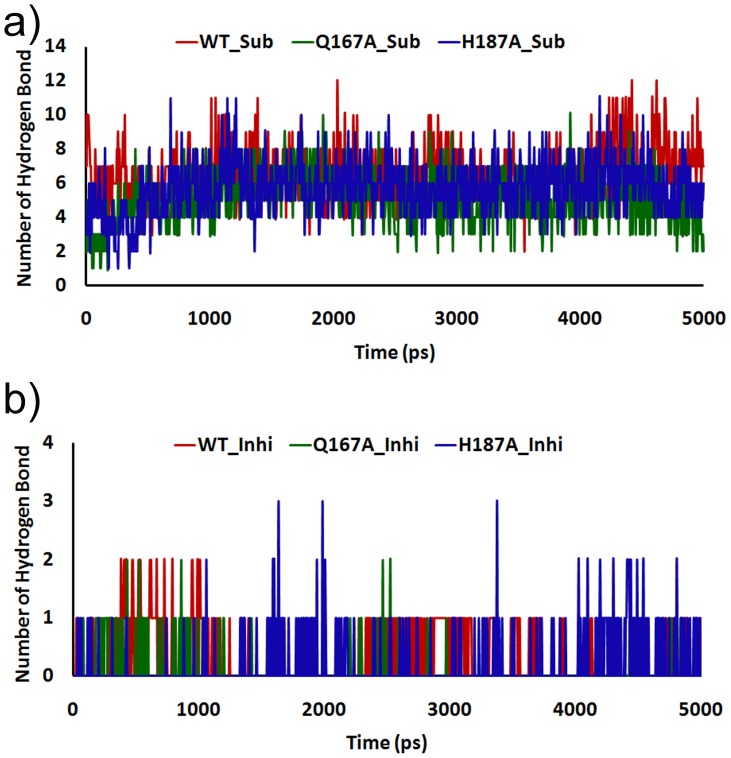
The total number of intermolecular hydrogen bond interactions exhibited by NAD^+^ and inhibitor in complex with SIRT2 as a function of time.

Hydrogen bond (Hbond) plays a crucial role in stabilizing the complex such as protein-substrate/NAD^+^, protein-activator, and protein-inhibitor. Therefore, we analyzed the Hbond framework for mutated structures as well as WT, to determine the interactions between protein and small molecules. Hbond between SIRT2 and substrate/NAD^+^ and inhibitor was calculated based on the two criteria: (1) a proton donor (D) and acceptor (A) distance of 3.5 Å or less and (2) a D-H … A angle of 120^o^ or more. In WT and mutants, SIRT2 substrate/NAD^+^ complex have shown a well conserved Hbond with NAD^+^ in WT but in both mutations it shows a less number of Hbonds compared with WT. The WT_Sub complex have shown approximately 10–12 number of Hbond but in case of both mutations the numbers of Hbonds was relatively less which implies that the mutation of B-site was not able to contact with substrate/NAD^+^ or inhibitor. The number and percentage of Hbond occupation of each complex was evaluated and shown in Figure. 9. Taking into account all the simulation results shown above disclosed that the NAD^+^ occupied a suitable place only in WT and due to both mutations the NAD^+^ was not able orient in the correct binding pocket. This analysis showed that B-site mutation affects the NAD^+^ binding which leads to suppress of SIRT2 deacetylation process.

### Reasons for the Assembly and Disassembly of C-site

From the above analysis it was clear that the mutation in the B-site would affect the C-site. These changes will be responsible for the assembly and disassembly of C-site. In case of substrate/NAD^+^ binding there was a π-π interactions between the phenyl group of F96 and nicotinamide part of NAD^+^ but this interaction was absent in case of mutations (Figure. 8). In WT_Sub complex, initially 0.7 nm was found between phenyl group of F96 and NAD^+^, after 2 ns the distance was maintained at 0.5 nm throughout the simulations. In case of both mutations we observed a distance greater than the WT, which confirmed there was less chance for the formation of π-π stacking interactions between the phenyl ring of F96 and NAD^+^. This indicates that F96 moved away from its original position. In case of Inhibitor binding, WT shows a average distance of 1.4 nm but Q167A and H187A shows a distance of ∼0.5 which was less than WT.

**Figure 10:**
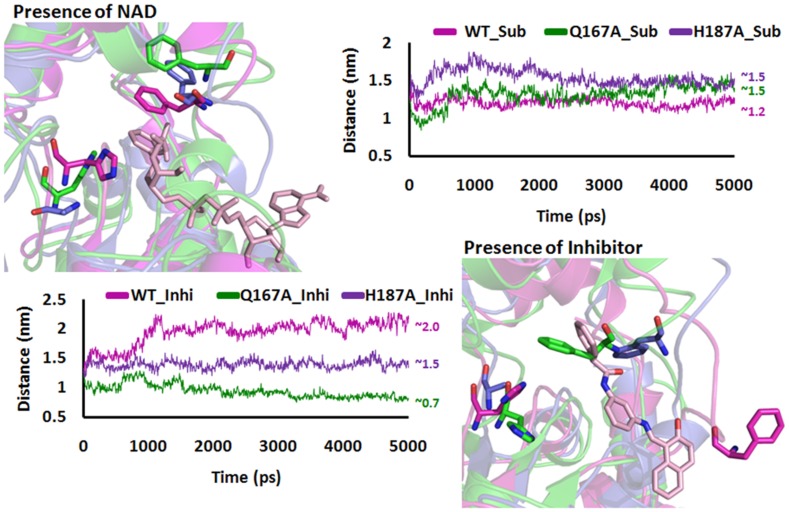
Time trace of Cα-Cα distance between F96 and H187 in WT was plotted as a function of time to highlight the difference in orientation of the conserved F96 in the L3.

To affirm the assembly and disassembly of C-site due to B-site mutations, the Cα distance was calculated between the residue H/A187 and F96, which was presented at the neck of the C-site. In presence of substrate, the WT shows a distance of 1.2 nm and maintained after 3 ns but in both mutations the distance was nearly 1.5 nm. In presence of inhibitor, the distance between these two residues was nearly 2 nm in WT but in Q167A and H187A the distance was less than 0.7 nm and maintained 1.3 nm, respectively (Figure. 10). The above results clearly insight that the mutations in B-site will affect the assembly of C-site, which was responsible for the SIRT2 deacetylation activity. To confirm the disassembly of C-site due to B-site mutations we compute the electrostatic potential map.

### Electrostatic Potential Surface Analysis

**Figure 11:**
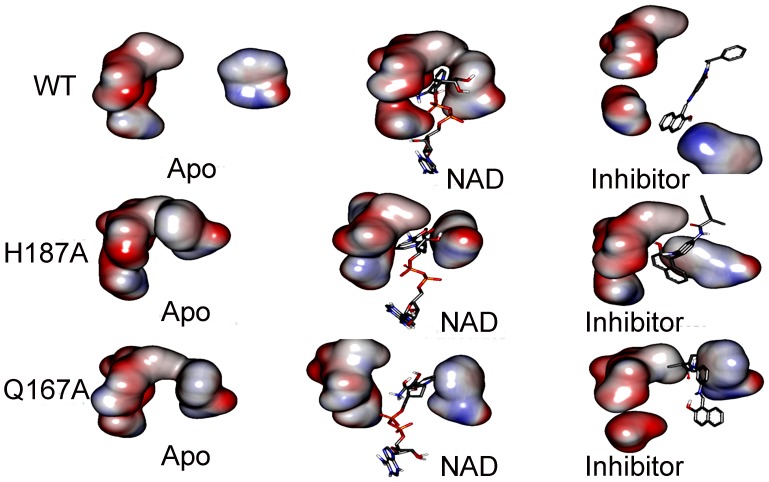
Electrostatic potential map indicates the assembly and disassembly of C-site in presence of NAD^+^ and inhibitor.

The electrostatic potential surface of protein serves as important driving force to direct the diffusion of small molecules into its active site as well as to stabilize the end point interactions between small molecule and protein. Thus we calculated SIRT2 APBS electrostatic potential surface [52] in presence of substrate/NAD^+^ and inhibitor for WT and mutants (Figure. 11). In the WT_Sub complex, the C-site has strong negative electrical property which can steadily hold the positive charge of nicotinamide in NAD^+^. The electrostatic potential of SIRT2 was retained in presence of inhibitor in WT but the assembly of C-site was disturbed by moving the F96 residue (2.07 nm) away from its original position (Figure. 11a). This reposition makes that the C-site not suitable for NAD^+^ binding by disassembly its shape. In both mutations (Q167A and H187A) the C-site was not able to assembly as in WT hence it was not able to process the deacetylation of NAD^+^ (Figure. 11) as effect as in WT. This confirms that the assembly of C-site was important for SIRT2 deacetylation process. From this result it was clearly visible that the assembly of C-site will be critical for the SIRT2 function. Hence, we suggest that this may be one of the reasons to reduce the SIRT2 activity which were experimentally proved by *Michael et al*.

### Conclusions

Sirtuins, NAD^+^-dependent histone deacetylases are interesting epigenetic regulators due to their actions on histones but also a regulator of signal transduction networks due to their non-histone substrates. A mutational study of SIRT2 has provided new insights into the molecular hindrances responsible for SIRT2 functions. Hence, MD simulations were performed to provide a structural basis to activate and inhibit the SIRT2 in presence of substrate/NAD^+^ and inhibitor. In this study, GROMACS package, GROMOS96 force field and explicit water molecules were used. The simulation results suggest the existence of important and functionally relevant differences between conformational dynamics of SIRT2. In WT due to NAD binding, large groove between large and small domains was narrowed which makes suitable place for NAD^+^ deacetylation reaction but due to mutations this groove was not able to narrow. NAD^+^ and inhibitor binding in WT bring F96 presents in L3 loop close and far away from H187, which make the assembly and disassembly of C-site, due to this process the nicotinamide deacetylation reaction was activated and inhibited, respectively. All the changes in WT are responsible for the formation of C-site cavity was absent in case of mutations. In summary, we have shown that the assembly and disassembly of C-site of NAD binding pocket by 5 ns time scale is related primarily to the presence of substrate/NAD+ and inhibitor. Therefore, we propose that any small molecules which increase the distance between F96 and H187 and make the disassembly of C-site as well as which can reduce the flexibility of L3 or shift F96 from its original place will be a good inhibitor for SIRT2.

## Supporting Information

File S1 **An archive containing the initial docking strucutre of WT and NAD, WT and Inhibitor, Q167A and NAD, Q167A and Inhibitor, H187A and NAD, and H187A and Inhibitor.**
(ZIP)
